# Botulinum toxin type A and acupuncture for masticatory myofascial pain: a randomized clinical trial

**DOI:** 10.1590/1678-7757-2020-1035

**Published:** 2021-06-04

**Authors:** Giancarlo DE LA TORRE CANALES, Mariana Barbosa CÂMARA-SOUZA, Rodrigo Lorenzi POLUHA, Cassia Maria GRILLO, Paulo César Rodrigues CONTI, Maria da Luz Rosário de SOUSA, Renata Cunha Matheus RODRIGUES GARCIA, Célia Marisa RIZZATTI-BARBOSA

**Affiliations:** 1 Universidade de São Paulo Faculdade de Odontologia de Bauru Departamento de Prótese BauruSP Brasil Universidade de São Paulo, Faculdade de Odontologia de Bauru, Departamento de Prótese, Bauru, SP, Brasil.; 2 Universidade Estadual de Campinas Faculdade de Odontologia de Piracicaba Departamento de Prótese e Periodontia PiracicabaSP Brasil Universidade Estadual de Campinas, Faculdade de Odontologia de Piracicaba, Departamento de Prótese e Periodontia, Piracicaba, SP, Brasil.; 3 Universidade Estadual de Campinas Faculdade de Odontologia de Piracicaba Departamento de Saúde Coletiva PiracicabaSP Brasil Universidade Estadual de Campinas, Faculdade de Odontologia de Piracicaba, Departamento de Saúde Coletiva, Piracicaba, SP, Brasil.; 4 UNINGA Departmento de Odontologia MaringáPR Brasil UNINGA, Departmento de Odontologia, Maringá, PR, Brasil.

**Keywords:** Botulinum toxin, Acupuncture, Myofascial pain, Temporomandibular disorders, Chronic pain

## Abstract

BoNT-A has been widely used for TMD therapy. However, the potential benefits compared to dry needling techniques are not clear.

**Objective:**

this study aimed to compare the immediate effects of botulinum toxin type A (BoNT-A) injections and Acupuncture in myofascial temporomandibular disorders (TMD) patients.

**Methodology:**

54 women were divided into three groups (*n*=18). AC patients received four sessions of traditional acupuncture, being one session/week during 20-min. BoNT-A patients were bilaterally injected with 30U and 10U in masseter and anterior temporal muscles, respectively. Moreover, a control group received saline solution (SS) in the same muscles. Self-perceived pain was assessed by visual analog scale, while pressure pain threshold (PPT) was verified by a digital algometer. Electromyographic evaluations (EMG) of anterior temporal and masseter muscles were also measured. All variables were assessed before and 1-month after therapies. The mixed-design two-way repeated measures ANOVA and Tukey’s post-hoc tests were used for analysis, considering a=0.05.

**Results:**

Self-perceived pain decreased in all groups after one month of therapy (*P*<.001). BoNT-A was not better than AC in pain reduction (*P*=0.05), but both therapies were more effective in reducing pain than SS (*P*<0.05). BoNT-A was the only treatment able to improve PPT values (*P*<0.05); however, a severe decrease of EMG activity was also found in this group, which is considered an adverse effect.

**Conclusion:**

after one month of follow-up, all therapies reduced the self-perceived pain in myofascial TMD patients, but only BoNT-A enhanced PPT yet decreased EMG.

## Introduction

Myofascial pain (MFP) is a disorder characterized by localized muscle tenderness, regional pain, and limited range of motion.^1^ It is the most typical cause of persistent regional pain, such as back and shoulder pain, tension-type headaches, and facial pain.^2^ Furthermore, it is a common condition in dentistry with prevalence from 10% to 68% among subjects with temporomandibular disorders (TMD).^3^

Masticatory myofascial pain (MMFP) has a complex pathogenesis expressed by a multifactorial etiology, which led to the proposal of numerous conservatives, reversible, and minimally invasive therapies to treat this condition.^4^ The needling technique is a minimally invasive therapy widely used for MMFP and it can be classified as an injection technique (IT), often referred to as “wet needling,” and dry needling (DN). While the IT deliver pharmacological agents, e.g., anesthetics, botulinum toxins or other agents, with needles,^5^ the DN consists in the insertion of thin monofilament needles, as the ones used for acupuncture practice, without any injectate.^6^

Acupuncture is a therapeutic method of the traditional Chinese medicine which differs from conventional DN techniques since needles are not inserted just in the painful region. Its antinociceptive effects^7^ include immediate reduction in local, referred, and widespread pain,^8^ and reduction in peripheral and central sensitization.^5^ Although a recent randomized clinical trial reported pain reduction of 84% after one month of treatment – concluding that acupuncture was effective for MMFP pain^9^, available systematic reviews did not find further advantages in the use of acupuncture for MMFP over other treatments such as oral appliances, behavioral therapy, and/or pharmacotherapies.^10,11^ These controversies might be due to several methodological shortcomings, leading to inconclusive results, which expose the need for high quality studies comparing the efficacy of acupuncture with other treatments.

Botulinum toxin type A (BoNT-A) is an FDA-approved treatment for some pain disorders (as dystonia and migraine), becoming one of the most popular IT used to control MFP.^4^ Animal studies have demonstrated that peripheral injections of BoNT-A have analgesic effects on pain stages by inhibiting the release of nociceptive mediators (peripherally and centrally), mechanism independent of its neuromotor effect.^12,13^ Based on this data, BoNT-A has been used as an off-label treatment to control MMFP. Moreover, a few well-designed clinical trials^14,15,16^ have demonstrated the superiority of this substance over placebo, but not over conservative treatments like oral appliances.^15^ Besides, a prospective study showed that BoNT-A injections in the masticatory muscles are also effective in reducing MMFP and tension-type headache, recommending this therapy for muscle pain.^17^ Conversely, the lack of consensus on the effects of BoNT-A is due to the number of low-quality studies available, and especially to the post-injection adverse effects in muscle and bone tissues, being the reason why its benefits for MMFP remain unclear.^18,19^

Studies comparing DN techniques with BoNT-A for myofascial TMD pain are scarce, and no previous study compared these treatments with an injection placebo group.^20-22^ Moreover, systematic reviews were inconclusive about the effectiveness of needling therapy, since it was not possible to determine if the technique (dry or wet) or the injectate were responsible for the improvements.^23^ Therefore, this study aims to compare the immediate effects of BoNT-A injections and acupuncture therapy in myofascial TMD patients.

## Methodology

### Experimental design

This randomized single-blinded controlled clinical trial, conducted following the Helsinki Declaration, was approved by the local Research Ethics Committee (CAAE: 22953113.8.0000.5418) and by the Brazilian Registry of Clinical Trials (ReBEC RBR-2d4vvv). All individuals were informed about the research purposes and signed an informed consent form.

The sample size estimation was based on the average pain scores of previous studies,^14,24^ and it was performed by using the G*Power 3.1.9.2 software (Düsseldorf, Germany). The following parameters were considered: a) test power of 0.9; b) 0.05 significance level; c) effect size of 0.4. Considering these standards, 15 participants per group would be sufficient to detect statistically significant differences. However, considering possible dropouts, 20% was added in each group. Thus, the final sample size comprised 54 individuals, which were randomly divided into three groups: acupuncture (*n*=18); BoNT-A (*n*=18); and saline solution (SS) (*n*=18), as a negative control group. For this allocation, a software was used (https://random-allocation-software.software.informer.com/2.0/) and the sequence was sealed in an opaque envelope, which was operated by a researcher not involved in other procedures of this study. The investigator assessing the outcomes was masked to the treatment assignments.

**Figure 1:**
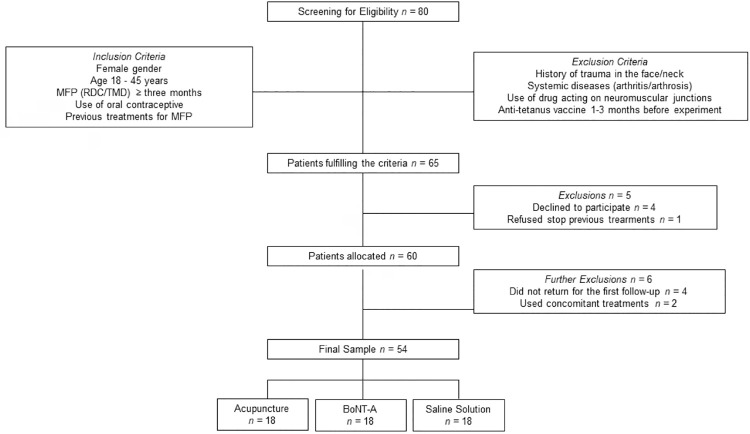
Flowchart of participants enrollment

The sample was obtained from women seeking for TMD treatment at the Piracicaba Dental School, University of Campinas, Piracicaba, Brazil, from 2014 to 2016. Patients had to be female with MMFP, with general good health, be aged between 18 and 45 years, with complete dentition, ongoing conservative treatment for at least three months without 30% of pain improvement and be using oral contraceptives in order to be included in the study. Subjects with systemic diseases (arthritis, arthrosis, diabetes), uncontrolled hypertension, neurological disorders, positive history of trauma in the orofacial and neck area, with dental pain, with self-reported sleep bruxism, and taking any medication for pain control were excluded from the recruitment (Figure 1). The diagnostic of MMFP was based on a clinical examination performed according to the official Portuguese version of the Research Diagnostic Criteria for Temporomandibular Disorders (RDC/TMD) – Axis I,^25^ by two calibrated raters (Kappa coefficient = 0.80). To achieve the 54 volunteers included in the study, 80 female subjects were screened for eligibility. Considering the inclusion and exclusion criteria, 65 participants were suitable to participate. However, before allocation, five individuals were excluded since they refused to participate (*n*=4) or to stop previous therapies (*n*=1). Six additional exclusions occurred due to lack of compliance, leading to a final sample of 18 volunteers per group (Figure 1).

### Therapies

#### Acupuncture

Acupuncture group received four sessions of traditional acupuncture, one session per week, with 20-min each for one month. The following points were selected for this therapy: LI4 (Hegu), LI11 (Quchi), SI19 (Tinggong), LR2 (Xingjian), GB20 (Fengchi), GB21 (Jianjing), GB34 (Yanglingquan), BL2 (Zanzhu), CV23 (Lianquan), and TE23 (Sizhukong).^24^ Disposable, sterile, individually packed, stainless steel needles (Huan Qiu; Suzhou Huanqiu Acupuncture Medical Appliance Co. Ltd., Suzhou, China) were used for this purpose by a acupuncturist. For facial points, needles had 0.22 mm of diameter and 13 mm length, and 0.25 mm diameter and 30 mm length for distal points. The needles were manually inserted and rotated in both clockwise and counterclockwise directions, until the patient related the sensation on needle site or along the meridian.

#### Botulinum toxin type A (BoNT-A)

**Figure 2:**
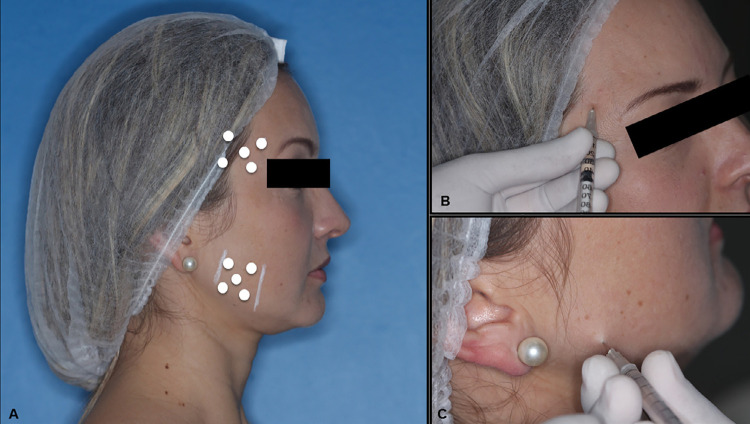
A, marked points for BoNT-A injection. B, temporal muscle injection; C, masseter muscle injection

BoNT-A (100 U; Botox, Allergan, Irvine, California, CA, USA) was reconstituted using non-preserved sterile saline solution 0.9%. A single bilateral injection was applied in the masseter and anterior temporalis muscles using 30U and 10U of BoNT-A, respectively^15^ by a calibrated researcher, distributed in five sites for each muscle. It was used a 1 mL syringe with a 30-gauge and 13 mm needle. Briefly, for the masseter, the injected sites were in the inferior part of the muscle (on mandibular angle), 5 mm apart from each other. For the anterior temporal muscles, sites were determined according to the functional test, considering the most prominent part, and must be 1 cm external to the eyebrow and 5 mm apart among them (Figure 2). Injections were performed bilaterally regardless the patient had pain in only one side of the face.

This injection technique consisted in inserting the needle into the soft tissue until reaching the bone; then, the needle was slightly moved to place the tip inside the muscle. Before injection, a careful aspiration was performed to avoid a possible intravascular administration.

#### Saline Solution (SS)

SS (NaCl 0.9%) was bilaterally injected into the same muscles and sites, following the same protocol and doses, as described for BoNT-A injections. Injections of BoNT-A and SS were performed in a single appointment by the same trained clinician, who was blinded to the treatment assignment.

## Outcomes

### Self-perceived pain: Visual Analog Scale (VAS)

VAS is a 100 mm horizontal line, anchored by the words “no pain” at the left end, and “worst pain imaginable” at the right end. Participants were instructed to mark a line at any point, representing the level of current, worst, and average pain of the last month.

### Pain sensitivity: Pressure Pain Threshold (PPT)

PPT was assessed by a digital algometer (Kratos DDK-20; São Paulo, Brazil) with 1 cm^2^ circular flat rod, for the bilateral evaluation of the masseter and anterior temporal muscles. Patients were instructed to indicate the moment when the pressure became painful. They were sat in a chair with the Frankfurt plane parallel to the ground, and muscles should be relaxed. The circular flat rod was perpendicularly pressed to the surface skin at a 0.5 kg/cm^2^ rate, following the sequence: right anterior temporal, right masseter, left masseter, and left anterior temporal muscles. After a five-minutes rest, the pressure was applied again, as follows: left anterior temporal, right anterior temporal, left masseter, and right masseter.

## Electromyographic assessment

The bilateral EMG signals of the anterior temporal and the superficial masseter muscles were recorded by the ADS 1200 device (Lynx Electronic Technology Ltd, Sao Paulo, Brazil), which has eight channels, adjusted gain of 1e16,000, band-pass filter of 20e500 Hz and sampling frequency of 2000 Hz per channel. A circular passive bipolar Ag/AgCl double electrode with 1 cm interelectrode distance was used (Hal Ind. Com. Ltda, Sao Paulo, Brazil).

Before the recordings, the volunteers’ skin was cleaned with cotton and 70% alcohol, and a function test was performed to identify the center of the muscle venter, in which the electrodes should be fixed. The reference electrode was placed on the participants’ manubrium of the sternum. The electrical activity of each muscle was recorded in mandibular postural position (rest) and maximum volunteer contraction (MVC). Each activity was measured three times, for five seconds, with a two minute period of rest between them, in order to avoid fatigue. The MVC activity was obtained by requiring the patients to chew a piece of Parafilm M (American National Can, Chicago, IL, USA) that was placed bilaterally in the molar region. Participants were instructed to clench their jaw to the maximum possible extent, and to maintain the pressure for five seconds, as were verbally stimulated by the examiner.

Simultaneous signals were obtained by the software Lynx AqDados 7.02 (Lynx Electronic Technology Ltd, Sao Paulo, Brazil), and the root mean square (RMS) values were processed by the software Lynx AqD Analysis 7.0 (Lynx Electronic Technology Ltd, Sao Paulo, Brazil). The RMS values of each acquisition were considered as those obtained in the 2s and 4s-interval. The mean values of the three acquisitions (Rest and MI) were considered.

As the evaluations were carried out in different timepoints, an acetate plate was fabricated for each patient to standardize the algometer position and the electrodes’ placement between sessions. The acetate plate followed the anatomic reference lines (external angle of the eye, tragus of the ear, and external angle of the mandible), and was clipped where the algometer and electrodes were placed.

## Statistical analysis

All data for groups and periods were expressed as means ± standard deviation (SD) and were assessed for normal distribution with the Shapiro-Wilk test. A mixed-design repeated measures two-way ANOVA test was used to observe the difference among groups over time and within the group. The statistical analysis compared the results observed before the treatment (baseline) with those observed one month after the therapies. Moreover, the three groups were compared to verify a possible statistically significant difference among therapies. The ANOVA test was followed by post hoc Tukey’s test. All analyses were performed using SPSS for Windows (release 21.0, SPSS Inc.), with a 5% significance level.

## Results

Participants’ age did not differ among the three groups. acupuncture (mean age 30.3±6.9 years); BoNT-A (mean age 34.6±6.5 years); and SS (mean age 30.8±6.9 years) (*P*= .124).

### Self-perceived pain (VAS)

**Figure 3:**
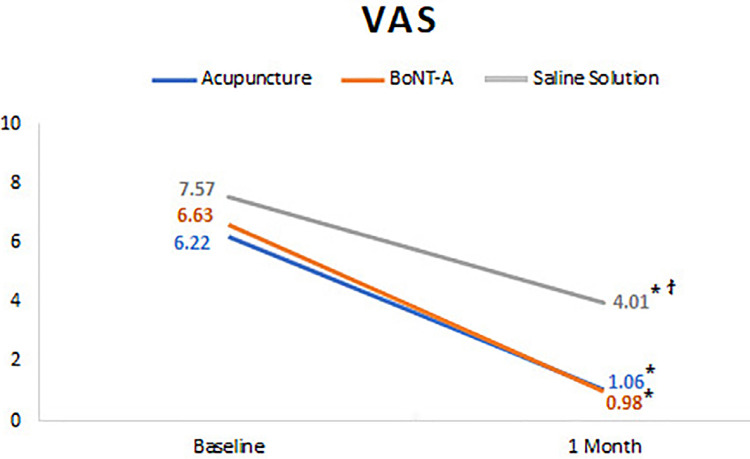
Self-perceived pain (VAS) values for each group, before and after treatments.

Self-perceived pain showed a significant decrease in all groups after one-month of therapy (*P*<.001). When comparing the different treatments, improvement in pain levels did not significantly differ between the acupuncture and BoNT-A groups (*P*>.05), but both groups presented a significant reduction on pain compared to the SS group (*P*<.001) (Figure 3).

ϯ represent significant differences among groups (P<0.05); * represent significant differences among time points (P<0.05).

### Pain sensitivity (PPT)

Considering the PPT values for the masseter muscles, the intragroup evaluation demonstrated that the acupuncture and SS groups did not show significant improvements (*P*=.359 and *P*=.220, respectively) after one-month of therapy. Notwithstanding, BoNT-A group presented significantly higher PPT values (*P*<.001) after one month of follow-up. Comparisons among groups, showed that acupuncture values did not differ from SS and BoNT-A (*P*=.751 and *P*=.123, respectively) groups. On the other hand, BoNT-A values were significantly higher than those obtained in the SS group (*P*=.006) (Table 1).

Furthermore, the PPT results for the anterior temporal muscles are in accordance with those achieve for the masseter muscles. Thus, intragroup evaluation showed no improvements on PPT values for the acupuncture and SS groups (*P*=.415 and *P*=.471, respectively), after one month of evaluation. Only the BoNT-A group presented statistically significant differences from baseline to the first-month follow-up (*P*<.001). Intergroup comparisons reveled that acupuncture values did not differ from SS (*P*=1.000) and BoNT-A (*P*=.111) values. However, BoNT-A values were statistically significant different just when compared with the SS group (*P*=.016) (Table 1).

### Electromyographic activity (EMG)

**Figure 4:**
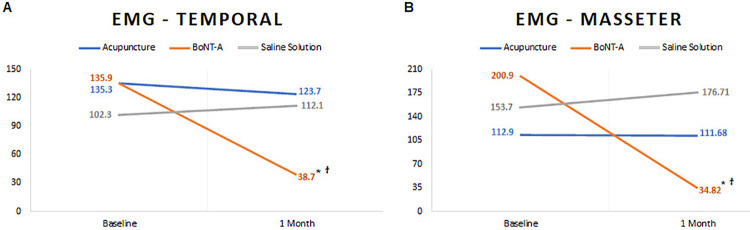
Root mean square scores (RMS μV) of maximum volunteer contraction for each group, before and after treatments. A, anterior temporal muscles mean values; B, masseter muscles mean values.

The EMG results for both masseter and anterior temporal muscles demonstrated that only volunteers in the BoNT-A group presented a significant reduction of the EMG activity one-month after the treatment (*P*<.001). Intergroup comparisons at the one-month follow-up showed a significant decrease in the masseter muscle activity in BoNT-A group compared to acupuncture (*P* =.020) and SS (*P* <.001), and these results were also found for the anterior temporal muscles (*P* <.001) (Figure 4).

ϯ represent significant differences among groups (P<0.05); * represent significant differences among time points (P<0.05).

## Discussion

The main findings of this study were that – after four weeks – all treatment groups (acupuncture, BoNT-A, and SS) were able to significantly reduce the self-perceived pain, without differences between acupuncture and BoNT-A, while both treatments were superior to the SS group. Moreover, considering the PPT values for both masseter and anterior temporal muscles, only BoNT-A group lead to a significant increase in PPT. Likewise, only patients treated with BoNT-A showed a significant reduction of the EMG activity in both studied muscles.

Pain is considered one of the most common reasons for a TMD patient seek treatment.^26^ All therapies used in this research were able to significantly decrease the self-perceived pain after one-month (*P*<.001). This was expected since the literature shows that the effectiveness of needling therapy in managing MMFP of the masticatory muscles does not necessarily depend on the needling type (dry or wet) or the injected substance, suggesting that the pain-reducing effect could be a consequence of the needle penetrating the skin.^23^

Comparing the treatments, improvement in pain levels did not significantly differ between the acupuncture and BoNT-A groups (*P*>.05). However, both groups presented a significant reduction on pain compared to the SS group (*P*<.001). A previous study showed that BoNT-A injection and dry needling yield similar satisfactory therapeutic outcomes regarding pain relief in patients with MMFP.^22^ Nevertheless, to the best of our knowledge, this is the first study comparing BoNT-A to acupuncture (not to a conventional dry needling) and including a placebo group. Considering that both BoNT-A and acupuncture have specific pain relief mechanisms, (reduction in peripheral and central sensitization), this can explain the improvement in the results achieved in comparison to SS group. Furthermore, two factors should be emphasized to explain the self-reported pain reduction: firstly, the placebo effect, in which the expectations generated by receiving a therapeutic approach is able to modulate pain perception, a mechanism known as placebo analgesia;^27^ secondly, the natural course of MMFP, which is generally favorable and with varying symptoms.^28,29^

Patients with MMFP usually present reduced PPT.^30^ PPT may be the most sensitive measure to detect endogenous pain inhibitory mechanisms.^31^ This measurement tests deep pain sensitivity, which is probably mediated by A-δ or C fibers; although pressure applied on the skin could reflect the pain sensitivity of both superficial and deep structures, the deep-tissue nociceptors mediate a major component of the pressure-induced pain during pressure algometry.^32^

Considering the PPT values for the masseter and anterior temporal muscles, the intragroup evaluation demonstrated that only BoNT-A group presented significantly higher PPT values (*P*<.001) at the follow-up. This result can possibly be explained by the exclusive antinociceptive mechanism of the BoNT-A. After BoNT-A injection, there is a temporary inhibition of pain neurotransmitter release at the pain site, reducing peripheral sensitization. Additionally, due to the BoNT-A axonal transport by A-δ and/or C fibers to the central nervous system,^33^ there is an indirect or direct reduction of central sensitization, hyperalgesia, and allodynia occurs (features usually associated with chronic pain, such as refractory MFP), through the reduction of the peripheral nerve over-activity, resulting in an increased pain threshold.^34^

Besides, some reasons might explain why acupuncture group did not show significant improvements in PPT: the short follow-up (one month), once a systematic review reported that dry needling treatments (such as acupuncture) may increase PPT immediately or until 12 weeks after the therapy;^35^ secondly, the type of acupuncture performed in this study (manual) may have influenced, as literature suggest that electroacupuncture is a technique more suitable to improve PPT.^36^

Between-groups comparisons on PPT values, BoNT-A treatment led to a higher increase in PPT values than in the SS group. This result is in accordance with a previous study that showed improvements of PPT after BoNT-A injection compared with the SS group in patients with MMFP.^37^ Notwithstanding, systematic reviews conclude that there is no consensus on the therapeutic benefits of BoNT-A on TMDs^38^ and that the efficacy-adverse effects ratio should be evaluated. Note that, in this study, BoNT-A improved all variables in a refractory MMFP population. These results reinforce that BoNT-A should not be the first option to MMFP treatment due to possible adverse effects, but it could be considered in patients that do not mitigate their pain with more conservative managements, fact that is corroborated by other studies.^14,15,39^

The EMG results for the masseter and anterior temporal muscles demonstrated that only patients treated with BoNT-A presented a significant reduction of the EMG activity one month after treatment (*P*<.001). Intergroup comparisons showed a significant decrease in muscle activity for both muscles in BoNT-A group when compared to acupuncture and SS groups. A reduction in EMG activity of masticatory muscles is expected after an intramuscular injection of BoNT-A once this toxin inhibits the release of acetylcholine at the neuromuscular junction of presynaptic motor neurons, reducing muscle activity.^39^ In fact, a temporary regional weakness is one of the most common adverse effects related to the use of BoNT-A in TMD treatment.^19^ A recent report also showed a significant reduction in maximum occlusal force in the BoNT-A group compared to SS and no injection groups.^40^

The occurrence and the intensity of these adverse effects are directly related to higher doses and repeated injections.^19^ In the present study, only a single injection of BoNT-A was used (30U in each masseter and 10U in each anterior temporal). Based on previous investigations,^15^ this dosage is able to reduce pain in MMFP patients, but it also allows the full recovery of muscle activity, which returned to normal EMG values after three months.^15^ Anyway, a reduction in the EMG activity of masticatory muscles is an adverse effect that must be considered; once acupuncture did not promote a significant change in EMG values, this could be considered an advantage of acupuncture over BoNT-A. Notably, the reduction of EMG values in BoNT-A groups is not responsible for decreasing subjective pain. Studies have demonstrated that BoNT-A presents an analgesic effect which is independent and precedes its neuromuscular effects. The EMG results in the acupuncture and BoNT-A groups confirms the disconnection between muscle electrical activity and muscle pain. Besides EMG reduction, patients receiving BoNT-A injections also reported adverse effects like edema and pain during injection, being the last also reported by the SS group. Conversely, self-reported adverse effects for the acupuncture group comprised itching and reddening of the skin, without pain symptoms nor edema.

These results suggest that all studied needling therapies (acupuncture, BoNT-A, and SS) are effective after one month of follow-up in reducing the self-perceived pain in patients with refractory MMFP; and, that BoNT-A seems to be superior due to the improvement in PPT values. Nevertheless, caution is necessary when judging these findings, since some limitations should be considered. Selecting only women as the study population hinder our results to be generalized to male patients. However, it was necessary once MMFP is more prevalent in this gender. Even though the 1-month follow-up is a restricted time of evaluation, our main objective was to assess the immediate effects of the proposed treatments; however, studies considering longer periods of evaluation should be performed. Finally, the effects of the proposed therapies on the psychosocial status of myofascial TMD patients should be also evaluated, considering that psychosocial variables generally act as chronification factors for TMDs.

## Conclusion

After one month of follow-up, all therapies reduced the self-perceived pain in patients with MMFP. BoNT-A was not superior to acupuncture in pain reduction, but both were superior to SS; moreover, BoNT-A was the only treatment able to improve PPT values. However, only patients treated with BoNT-A reduced the EMG activity in the injected muscles which should be considered as an adverse effect.

**Table 1:** Mean and standard deviation (SD) of pain pressure threshold (kg/cm-2) for each group, before and after treatments

Group/PPT-Muscle	Baseline	1 Month
Temporal		
Acupuncture	0.67 (±0.27)^Aa^	0.73 (±0.19)^Aa^
Botulinum Toxin A	0.54 (±0.22)^Aa^	0.92 (±0.29)^Ab^
Saline Solution	0.61 (±0.23)^Aa^	0.66 (±0.33)^Aa^
Masseter		
Acupuncture	0.67 (±0.23)^Aa^	0.72 (±0.18)^Aa^
Botulinum Toxin A	0.53 (±0.19)^Aa^	0.88 (±0.25)^Ab^
Saline Solution	0.57 (±0.17)^Aa^	0.64 (±0.25)^Aa^

Different uppercase letters represent significant differences among groups (P<0.05); different lowercase letters denote significant differences among assessment time points (P<0.05); PPT: Pressure pain threshold; kg/cm^2^=Kilogram per square centimeter.
